# Failure of the Interference Tenodesis Screw After Distal Bicep Tendon Repair With a Suture Button Technique: A Report of Two Cases

**DOI:** 10.7759/cureus.13779

**Published:** 2021-03-09

**Authors:** Daniel Fletcher, Francis J Sirch, Connor Fletcher, Pedro Beredjiklian, Jonas Matzon

**Affiliations:** 1 Division of Hand Surgery, Rothman Orthopaedic Institute, Philadelphia, USA; 2 Department of Orthopaedics, Rothman Orthopaedic Institute, Philadelphia, USA

**Keywords:** tendon reconstruction, bicep tendon, tendon rupture, endobutton, interference screw

## Abstract

Distal bicep tendon rupture is an uncommon form of bicep rupture which is typically seen in middle-aged men. We identified two cases in which the distal bicep tendon rupture was repaired with an EndoButton™ (Smith and Nephew, Watford, UK) and interference screw with preservation of the EndoButton™​ but a failure of the interference screw. This report highlights the addition of a FiberWire^®^ (Arthrex, Inc., Naples, Florida, USA) construct to secure the interference screw from backing out and emphasizes the EndoButton™ as the primary biomechanical anchor in maintaining a successful distal bicep tendon repair. We question the necessity of both the interference screw and EndoButton™ in the fixation of the distal bicep tendon and recommend that securing the interference tenodesis screw with an additional FiberWire^®^ may provide a more secure fixation of the screw as compared to traditional approaches.

## Introduction

Innovation in surgical approach and fixation after distal biceps tendon ruptures has been prevalent in the past two decades with the development of a modified two-incision technique, suture anchors, cortical buttons, and interference screws [1]. Biomechanical studies have demonstrated that cortical button fixation has the highest load to failure compared to other fixation techniques. There is evidence that the addition of an interference tenodesis screw can help maintain strength, reduce gap formation, and reduce the motion of the fixation site [2,3]. The purpose of this study is to report two cases of a successful distal biceps tendon repair with a suture button technique despite the loss of fixation of the interference tenodesis screw.

## Case presentation

Case 1

A 51-year-old man with a past medical history significant for hypertension and coronary artery disease presented with left elbow pain after forcefully shutting a car door. He described hearing and feeling a “snap” like a rubber band. Physical examination revealed proximal migration of the left biceps muscle mass, ecchymosis around the elbow, abnormal biceps hook maneuver, and inability to palpate the distal biceps tendon. He had increased discomfort with resisted forearm supination. X-ray evaluation was normal.

The patient elected for surgery, and an anterior single incision distal biceps tendon repair utilizing a BicepsButton™ (Arthrex, Inc., Naples, Florida, USA) and 7 mm x 10 mm BioComposite™ interference tenodesis screw (Arthrex, Inc., Naples, Florida, USA) was completed without complication. The Arthrex distal biceps repair using the BicepsButton™ and tension-slide technique surgical technique guide was followed, except that a 7.5 mm reamer was used in place of the 8.0 mm described in the technique guide [4]. The positioning of the suture button, integrity of the radius, and the placement of the tenodesis screw were visually and fluoroscopically confirmed intra-operatively. The wound was irrigated, closed, and a posterior long-arm splint was applied. Two weeks post-operatively, the patient was placed in a removable splint and instructed on a physician-guided passive range of motion home therapy program.

**Figure 1:**
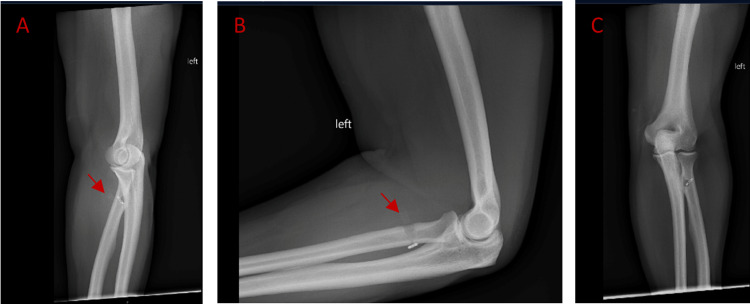
Four-month post-operative image (A) Oblique view demonstrating proximal migration of the tenodesis screw (red arrow) with intact EndoButton™ repair.  (B) Lateral view with visible proximal migration of interference screw (red arrow), EndoButton™ on the posterior cortex. (C) Anterior-posterior view illustrating the intact fixation of the EndoButton™ aligned with the central aspect of the radial tuberosity.

At 3.5 months postop, the patient reported numbness to the dorsum of his index and long fingers. Examination revealed an intact distal biceps tendon, a normal biceps hook maneuver, full range of motion, decreased sensation over the superficial radial nerve distribution, with normal strength of muscles innervated by the radial, median, and ulnar nerve. X-ray evaluation at 3.5 months postop follow-up demonstrated proximal migration of the tenodesis screw but intact suture button on the posterior cortex of the radius (Figure 1). 

**Figure 2:**
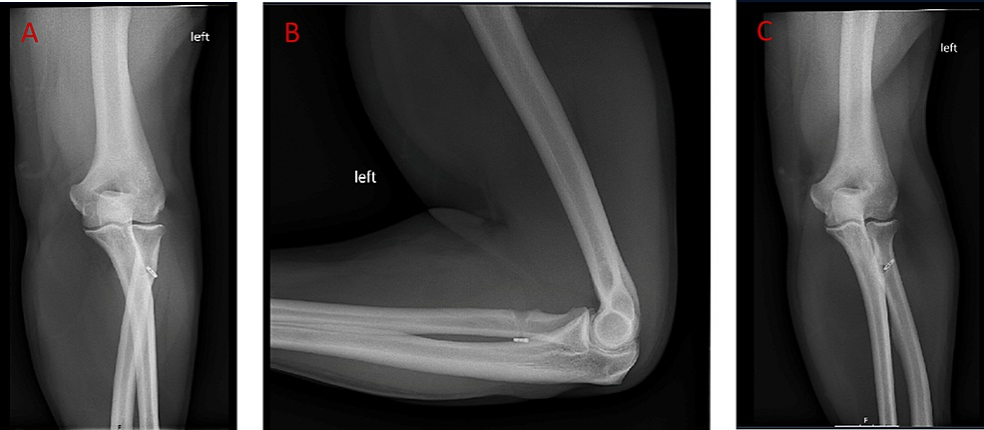
Fifteen-month post-operative image (A) Anterior-posterior pronation view with good EndoButton™ alignment.  (B) Lateral view demonstrating intact fixation of the EndoButton™ on the posterior cortex of the radius and complete tenodesis screw resorption. (C) Anterior-posterior supination view with good EndoButton™ alignment.

Final evaluation at 15 months postop revealed an intact distal bicep tendon, normal biceps hook maneuver, normal sensation, full strength, radiographic confirmation of tenodesis screw resorption, and continued full-duty work as a sheriff (Figure 2).

Case 2

A 52-year-old man presented with left elbow pain and deformity after lifting a toilet into a dumpster. He felt a tear and noted difficulty lifting.

Physical examination revealed proximal migration of the left biceps muscle mass approximately 6-8 cms, ecchymosis, abnormal biceps hook maneuver, and inability to palpate the distal biceps tendon. He had increased discomfort with resisted forearm supination with the elbow at 90 degrees and 0 degrees. X-ray evaluation demonstrated mild degenerative arthritis and calcification at the tip of the olecranon.

The patient elected for surgery, and an anterior single incision distal biceps tendon repair utilizing a BicepsButton™ and 7 mm x 10 mm BioComposite™ interference tenodesis screw was completed without complication. The identical surgical technique described in Case 1 was followed for Case 2. Ten days post-operatively, he was placed in a removable splint and instructed on a physician-guided passive range of motion home therapy program.

**Figure 3:**
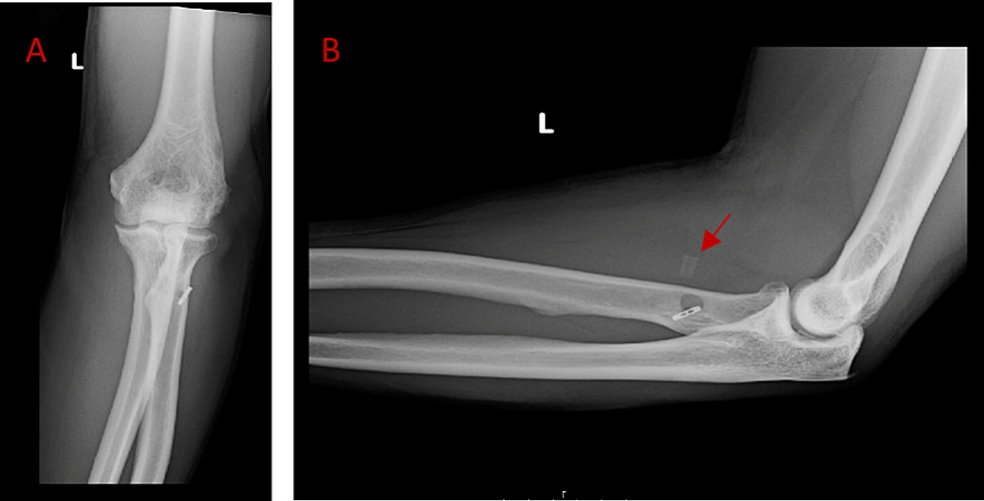
Three-month post-operative image (A) Anterior-posterior view with intact initial EndoButton™ fixation. (B) Lateral view demonstrating central alignment of the EndoButton™ on the posterior cortex of the radius, and proximal migration of the interference screw (red arrow).

At nine weeks postop, the patient reported fullness and numbness around his incision. Examination revealed an intact distal biceps tendon, a normal biceps hook maneuver, normal sensation, and normal motor strength. X-ray evaluation demonstrated the proximal migration of the tenodesis screw but an intact suture button abutting the posterior cortex of the radius (Figure 3).

**Figure 4:**
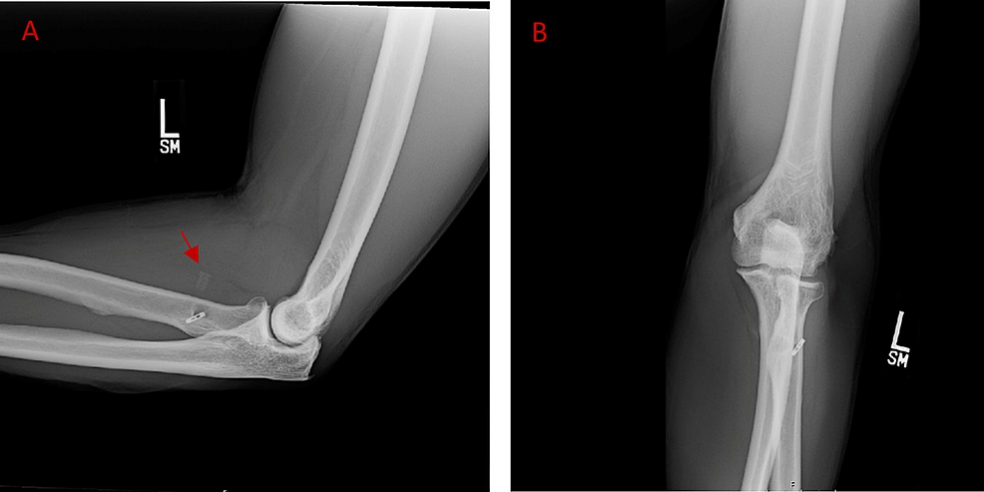
Eight-month post-operative image (A) Lateral view of fixation of the EndoButton™ with proximal migration and partial resorption of the interference screw (red arrow). (B) Anterior-posterior supination view confirming alignment of the EndoButton™ on the posterior cortex of the radius.

Clinical evaluation at eight months postop revealed an intact distal biceps tendon, normal biceps hook maneuver, normal sensation, full strength, radiographic confirmation of the tenodesis screw resorption, and return to his occupation in construction (Figure 4).

## Discussion

Failure of surgical fixation is rare in distal biceps tendon repair, with a recent meta-analysis reporting a rate of 0.6-2% for the varied fixation techniques [5-7]. However, complications have been well documented for both single-incision and double-incision surgical approaches. A literature review suggests an overall complication rate between 21-30%, with a higher complication rate with the double-incision approach [1,5,8]. The most common complication with a single-incision approach is a higher rate of lateral antebrachial cutaneous nerve palsy, while double-incision approaches report higher rates of posterior interosseous nerve palsy, heterotopic bone formation, and higher reoperation rates [5]. Watson et al. published a 2014 review suggesting a single-incision approach with bone tunnels and cortical buttons had the lowest rate of complications. However, Kodde et al. published a 2016 review suggesting fewer complications after a double-incision approach with bone tunnel fixation [1,6]. Lastly, a 2018 prospective study by Matzon et al. reported early post-operative complications of 44.6% in a one-incision distal biceps repair cohort compared to 15% in a two-incision repair cohort [9]. Cumulatively, these findings illustrate that current evidence has yet to reach a consensus on surgical approach and fixation technique to provide optimal outcomes.

**Figure 5:**
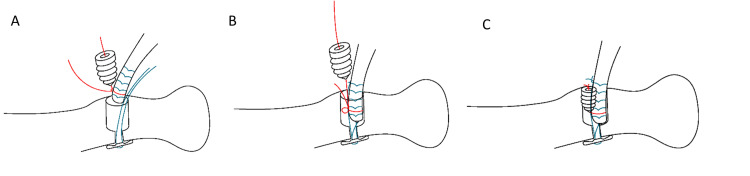
Modified suture technique (A) Blue FiberLoop® whip stitch secures bicep tendon and is passed through EndoButton™, additional red FiberWire® is tied to the distal tendon while one limb is passed through the interference screw. (B) Bicep tendon is advanced into the radius and secured to tendon proximally with blue FiberWire®. (C) Interference screw is inserted and held in place by tying red FiberWire®.

We report two cases with failure of the interference tenodesis screw but with preservation of the suture button construct still allowing for successful distal biceps tendon repair. In our cases, the ruptured biceps tendon was repaired using a SpeedWhip™ technique (Arthrex, Inc., Naples, Florida, USA) with a number 2 FiberLoop® (Arthrex, Inc., Naples, Florida, USA), BicepsButton™, and BioComposite™ interference screw [4]. Intraoperative direct visualization and fluoroscopy were used to confirm radius integrity and suture button and interference tenodesis screw placement. The FiberLoop® was sutured to the biceps tendon just anterior to the radial cortex while maintaining tension on the suture button and biceps tendon repair, creating an open loop of FiberWire® (Arthrex, Inc., Naples, Florida, USA). The interference screw was then inserted, and the remaining limb of FiberLoop® was tied over the screw [4]. The open FiberWire® loop sutured anterior to the radial cortex allows the backing out of the interference screw with early forearm rotation, elbow flexion, and elbow extension. Recently, our distal biceps tendon repair has been modified by the addition of a FiberWire® to the distal biceps tendon. One limb of this deep intraosseous FiberWire® is passed through the interference screw, the screw is inserted, and the additional FiberWire® limp is tied over the screw preventing it from backing out (Figure 5). We have not seen the interference tenodesis screw malposition since this technique modification. A prior case report of interference screw failure and resultant EndoButton™ loosening provided guidelines that the reamer should be at least 0.5 mm to 1 mm larger than the intended screw diameter to accommodate for tendon volume, target the radial tuberosity centrally and perpendicularly to ensure a uniform cortical wall, and maintain constant tension on the tendon limb to promote tendon engagement during screw placement [10]. Despite providing valuable suggestions to improving tendon-screw engagement, it remains unclear if the loosening of the interference screw was the primary cause of the case’s fixation failure. Additionally, cases in which the tenodesis screw has failed while the suture button construct remains intact have yet to be reported in the literature. 

These cases raise the question of whether the interference tenodesis screw is necessary for distal biceps repair with a cortical button. Biomechanical studies demonstrate that the cortical button provides the most load to failure, followed by suture anchor repair, bone tunnel, and interference screw [3]. Mazzocca et al.’s biomechanical study demonstrated that all fixation techniques experienced displacement with early post-operative phase cyclic loading and proposed a combined fixation method utilizing the cortical button and interference screw to limit early cyclical movement [3,11]. Sethi et al. report that pistoning of the tendon occurs with elbow motion and theorized that early gap formation that occurs with the cyclical movement could inhibit direct tendon healing. However, their study found no difference in functional outcomes with the addition of the interference screw to the cortical button [2]. Heinzelmann et al. demonstrated that a soft tissue button and interference screw allow accelerated rehabilitation and early return to function, but do not discuss if this would occur with cortical button alone [12]. Additionally, the cortical button with interference screw and cortical button by itself has been shown to provide similar outcomes and complications as compared to a suture anchor [13-15]. 

Post-operative distal biceps tendon re-rupture is low, with re-rupture rates after primary repair most prevalent within three weeks of the original repair [7,16]. Re-rupture can occur due to anchor-interference screw failure, tendon-suture interface failure, as well as mal-positioning of the fixation system [7]. Biomechanical studies support the cortical button as the strongest load to failure, and low re-rupture rates suggest that most surgical approaches adequately fixate the ruptured biceps tendon. Additionally, re-rupture has been suggested to result from poor patient education, with excessive eccentric forces introduced to the primary fixation. This suggests that most re-rupture is due, intentionally or inadvertently, to non-compliance [7,16]. Literature review provides no direct investigations comparing suture button repair alone with the combination of suture button and interference tenodesis screw. However, a direct comparison between suture button and interference screw has been reported, with load to failures ranging from 259 N - 439 N for the cortical button and 131 N - 294 N for the interference screw. Additionally, Arianjam et al. compared interference screw to interference screw and cortical button and demonstrated no significant difference between the two loads to failure strengths [2,3,17-19]. Lastly, Greenberg et al. compared the pull-out strength of three anchoring methods and revealed the cortical button was three times as strong as the bone bridge and twice as strong as the Mitek suture anchor (DePuy Mitek, Inc., Raynham, USA) [18]. If there are no significant changes in re-rupture rate nor improvement in complications nor patient outcomes, the utilization of both a suture button and interference screw could be resulting in more complicated fixation techniques and needlessly increased healthcare costs.

## Conclusions

Distal biceps tendon rupture is an uncommon form of biceps rupture, typically seen in middle-aged men. This case report highlights the addition of a FiberWire® construct to secure the interference screw from backing out and emphasizes the BicepsButton™ as the primary biomechanical anchor in maintaining a successful distal biceps tendon repair. Further investigation should pursue the necessity of both the interference screw and suture button in the fixation of the distal biceps tendon. However, we currently recommend securing the interference tenodesis screw with an additional FiberWire® to provide a more secure fixation of the screw than traditional approaches.
